# Grafting of a Single Donor Myofibre Promotes Hypertrophy in Dystrophic Mouse Muscle

**DOI:** 10.1371/journal.pone.0054599

**Published:** 2013-01-18

**Authors:** Luisa Boldrin, Jennifer E. Morgan

**Affiliations:** The Dubowitz Neuromuscular Centre, UCL Institute of Child Health, London, United Kingdom; University of Rome La Sapienza, Italy

## Abstract

Skeletal muscle has a remarkable capability of regeneration following injury. Satellite cells, the principal muscle stem cells, are responsible for this process. However, this regenerative capacity is reduced in muscular dystrophies or in old age: in both these situations, there is a net loss of muscle fibres. Promoting skeletal muscle muscle hypertrophy could therefore have potential applications for treating muscular dystrophies or sarcopenia. Here, we observed that muscles of dystrophic *mdx* nude host mice that had been acutely injured by myotoxin and grafted with a single myofibre derived from a normal donor mouse exhibited increased muscle area. Transplantation experiments revealed that the hypertrophic effect is mediated by the grafted fibre and does not require either an imposed injury to the host muscle, or the contribution of donor cells to the host muscle. These results suggest the presence of a crucial cross-talk between the donor fibre and the host muscle environment.

## Introduction

Regeneration of skeletal muscle is primarily mediated by the resident adult muscle stem cells [1]–[3]. Satellite cells are the principal muscle stem cells and the main source of muscle fibres (myofibres). In adult muscle, they are quiescent cells, located in niches between the basal lamina and sarcolemma of each fibre. However, following muscle injury, they become activated, proliferate and differentiate to repair or replace myofibres and by self-renewing they functionally reconstitute the muscle stem cell pool [4], [5]. Evidence of their enormous *in vivo* potential is given by the capacity of the few satellite cells associated with a single fibre [6], or a few hundred satellite cells isolated from fibres, to efficiently repair and regenerate host fibres after grafting in murine recipient muscles [6]–[9]. However, donor-derived muscle regeneration can be efficient only if the host satellite cell niche is preserved with concomitant functional impairment of the host satellite cells [9].

Moreover, muscle regeneration is highly dependent on the pathological status and age of the muscle environment. In advanced stages of neuromuscular degenerative disorders, for example in Duchenne muscular dystrophy (DMD), skeletal muscle becomes substituted by fibrotic, connective and adipose tissue, which hampers muscle regeneration [10], [11]. In the naturally-occurring genetic and biochemical homologue of DMD, the *mdx* mouse, exacerbation of the pathology produces similar tissue degeneration [12]. Muscle function is impaired within aged skeletal muscle where a concomitant gradual loss (sarcopenia) of muscle fibres and replacement of muscle with fibrotic tissue cause muscle atrophy and weakness, all features of aged muscle [13]. Moreover, wasting muscle syndrome (cachexia) is seen in patients with cancer, AIDS, and other severe chronic disorders [14].

A therapeutic intervention that specifically modulates skeletal muscle hypertrophy would potentially provide benefit to all these conditions. Restoration and improvement of muscle mass have been reported in muscles of mice in which IGF-1 was specifically overexpressed, making hypertrophic myofibres that were able to elude age-related muscle atrophy [15]. Myostatin, a protein that negatively-regulates muscle mass, also appears to be a crucial regulator of muscle mass, as mutations in its gene cause muscle hypertrophy [16]–[22]. Blocking the myostatin pathway has been suggested as a potential way of intervention, since systemic delivery of myostatin antagonists [23], or inhibitors, induces muscle growth [24]–[26].

The role of satellite cells in adult muscle maintenance, as opposed to regeneration, has been controversial [27]–[30], but recent data have highlighted a subpopulation of satellite cells responsible for muscle growth and routine maintenance [8]. How their contribution is triggered and regulated remains to be investigated. Interestingly, signals responsible for muscle growth may originate from the fibre itself [31], [32]. Shedding light on this key process is of fundamental importance in order to prevent muscle atrophy.

Here, starting from our experimental observation that engraftment of single fibres in myotoxin-injured muscles causes an increase in the size of the grafted muscles, we have further explored this phenomenon. We found that grafting of a single fibre is able to trigger a hypertrophic muscle effect even in uninjured *mdx* mouse muscles and the presence of the fibre itself is an essential requirement for this effect.

## Materials and Methods

### Host Mice and Muscle Injury

Breeding of mice and experimental procedures were carried out in the Biological Services Unit of University College London, Institute of Child Health, in accordance with the Animals (Scientific Procedures) Act 1986. Experiments were performed under Home Office licence.

Three-week-old *mdx* nude mice [33] were anaesthetised with hypnorm and hypnovel to irradiate their hindlimbs with 18Gy (at dose rate of 0.72Gy/minute) or isoflurane to inject 25 µl of 1.2% barium chloride (BaCl_2_) (Sigma, UK) into their *tibialis anterior* (TA) muscles. When single fibres were grafted in irradiated muscles, 10 µl of *Notechis scutatus* notexin (10 µg/ml) were injected into host muscles immediately prior to grafting one single fibre per muscle, to increase the incidence of donor satellite cell engraftment [6]. As analgesic after BaCl_2_ or notexin injections, vetergesic (50 µg/kg) was injected subcutaneously into the mice. As controls, either 25 µl of phosphate buffered saline (PBS) or 25 µl of Dulbecco’s modified Eagle’s medium (DMEM) (Invitrogen) was injected, as indicated in the experimental design.

### Donor Mouse Models

Adult (2–3 months old) genetically modified 3F-*nlacZ*-2E and β-actin-Cre:R26NZG (obtained from crossing a homozygote male β-actin-Cre (FVB/N-Tg(ACTB-cre)2Mrt/J) -a kind gift from Massimo Signore, UCL- with an homozygote female R26NZG (Gt(ROSA)26Sortm1(CAG-lacZ,-EGFP)Glh) (The Jackson Laboratory, USA)) mice were used as donors. β-galactosidase (β-gal) is expressed in all myonuclei in 3F-*nlacZ*-2E mice [34] and ubiquitously in all nuclei of β-actin-Cre:R26NZG mice [35], [36]. These two models allow us to identify either myonuclei alone, or all nuclei (including those outside myofibres) of donor origin, within grafted muscles.

### Donor Fibre and Satellite Cell Preparation

*Extensor digitorum longus* (EDL) muscles were isolated from donor mice as previously described [37], [38]. Briefly, after mice were killed by cervical dislocation, EDL muscles were carefully isolated from tendon to tendon under microscopic observation and digested in 2% collagenase type I (Sigma)/DMEM at 35°C for 70 minutes. Muscle fibres could then be easily separated under a stereo microscope by using heat-polished, pulled glass Pasteur pipettes. Fibres were serially washed to eliminate debris and other muscle components and only intact, clean myofibres were carefully selected. Some fibres were carefully transferred in a plate in DMEM and kept at 37°C for less than an hour before 4 µl of DMEM containing one fibre were grafted into the middle part of each host muscle by means of fine glass needle. In the experiments where satellite cells, rather than isolated fibres, were grafted, an aliquot of the fibre preparation was triturated to release satellite cells [7], [38], [39] and approximately 4 µl of DMEM-containing 400 of these cells was grafted into TA muscles of host mice by means of fine glass needle [7], [40]. Host mice were grafted 3 days after muscle injury and muscles were removed for analysis 4 weeks after grafting.

### Analyses of Grafted Muscles

At the time of harvesting, muscles were frozen in isopentane chilled in liquid nitrogen. Seven µm serial transverse cryosections were cut throughout the entire muscle. When grafted with donor single fibres or satellite cells, the presence of donor nuclei was evaluated by X-gal staining. Transverse sections serial to those containing X-gal stained nuclei were immunostained with P7 dystrophin antibody [41] and counterstained with 4′,6-diamidino-2-phenylindole (DAPI) fluorescent dye (Sigma, UK). The expression of myosin 3F-*nLacZ*-2E by dystrophin-positive fibres is evidence that the group of fibres was of donor origin [6], [7], rather than being host (revertant) [42], [43] fibres. Quantification of donor-derived nuclei and fibres was performed in the section with the highest number of donor-derived dystrophin-positive fibres [6], [7].

Analyses of muscle cross section area (CSA), number and myofibre area were performed on cryo-sections that had been stained with polyclonal laminin antibody (Sigma, UK) or with haematoxylin and eosin (H&E) [44]. Serial transverse sections were cut throughout the entire muscle and the largest transverse section was selected for analysis. Multiple images, captured at 10× magnification, from the selected section were assembled to give an image of the entire section and this was used for quantification of CSA and number and area of myofibres.

### Image Capture and Quantitative Analyses

Fluorescence and brightfield images were captured using a Zeiss Axiophoto microscope (Carl Zeiss, UK) and MetaMorph image capture software (MetaMorph software, USA).

Digitalization of images and quantification were performed with ImageJ (rsbweb.nih.gov/ij). Graph and figures were assembled using Photoshop CS2 software.

### Statistical Analyses

Results are reported as mean ± SEM from an appropriate number of samples, as detailed in the figure legends. Student’s t-test and Chi-squared test were performed using GraphPad software to determine statistical significance.

## Results

### Single Donor Myofibres Grafted into BaCl2-treated Host Muscles do not Contribute to Muscle Regeneration, but do Cause Muscle Hypertrophy

**Figure 1:**
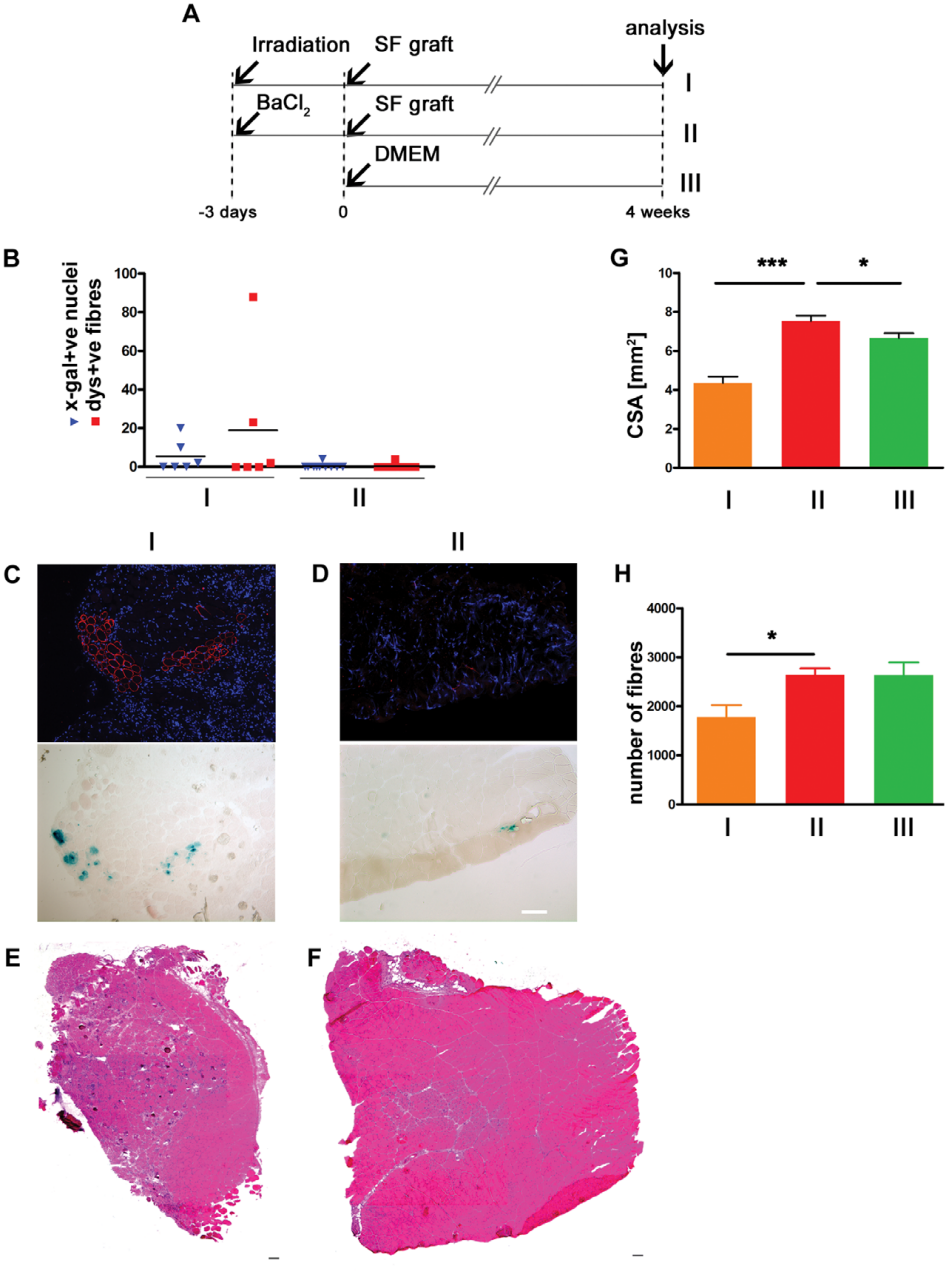
Single myofibres grafted into BaCl2-treated host muscles give rise to no donor-derived muscle formation but cause muscle hypertrophy. Single fibres (SF) isolated from a 3F-nlacZ-2E donor mouse were grafted into TA muscles that had been either irradiated 3 days before (n = 6) (A-I), or that had been BaCl2-injected three days before (n = 10) (A-II); as a control, DMEM was injected into untreated TA muscles (n = 10) (A-III). Donor-derived myofibres (identified by dystrophin expression and incorporation of X-gal positive myonuclei 4 weeks after grafting) were clearly observed in pre-irradiated grafted muscles (I), but to a trivial extent in BaCl2-injured grafted muscles (II) (B), as shown by representative pictures of dystrophin positive fibres with donor-derived myonuclei X-gal stained in serial sections (C and D respectively for I and II). H&E staining of whole transverse-sections from the largest middle part of grafted TA muscles highlighted the difference in size between muscles in I (E) and II (F). This difference was quantified in (G) showing that the cross-sectional area (CSA) of BaCl2-injured and SF-grafted muscles (II) was significantly bigger than in I and III. The number of fibres was not increased in II compared to control, but a loss of fibres was detected in I (H). Size bar = 100 µm. *p<0.05; ***p<0.0001.

As pre-modulation of host muscle is needed to promote donor satellite cell engraftment [45], and a single donor myofibre grafted in pre-irradiated host muscles generated donor-derived muscle [6], we wished to test if a different muscle modulation - BaCl_2_ that induces muscle degeneration and regeneration - could promote donor myofibre-mediated engraftment to the same extent. *Tibialis anterior* (TA) muscles of *mdx* nude recipient mice were injected as detailed in the experimental plan in Figure 1A. Donor-derived fibres were found in muscles pre-treated by either BaCl_2_ or irradiation and grafted with an isolated fibre. However, whilst donor-derived muscle fibres (ranging from 2 to 88) were found in 50% of the irradiated muscles, only 4 donor-derived fibres were found in 1 out of 10 host muscles that had been pre-treated with BaCl_2_ (Figure 1B, C, D). The hematoxilin and eosin (H&E) histological analyses revealed that, despite the negligible contribution to donor-derived muscle formation, the cross-sectional area (CSA) of muscles grafted following BaCl_2_ with one isolated fibre was larger than the CSA of muscles grafted after irradiation with an isolated fibre (Figure 1E, F, G). This is due to the progressive loss of host myofibres following irradiation (Figure 1H) [46]. Furthermore, the BaCl_2_-injected and grafted muscles were significantly greater in weight than the non-injured DMEM-injected muscles (Figure 1G). We found no obvious differences in the extent of fibrosis or adipogenesis in mouse muscles treated in the different ways. As we did not find a difference in the number of fibres between BaCl_2_-injured muscles injected with a myofibre in DMEM and non-injured muscles injected with DMEM alone (Figure 1H), we conclude that the donor fibre does not contribute to muscle regeneration in in BaCl_2_-treated muscles, but it induces a hypertrophic effect.

### BaCl2 is not the Cause of Muscle Hypertrophy

**Figure 2:**
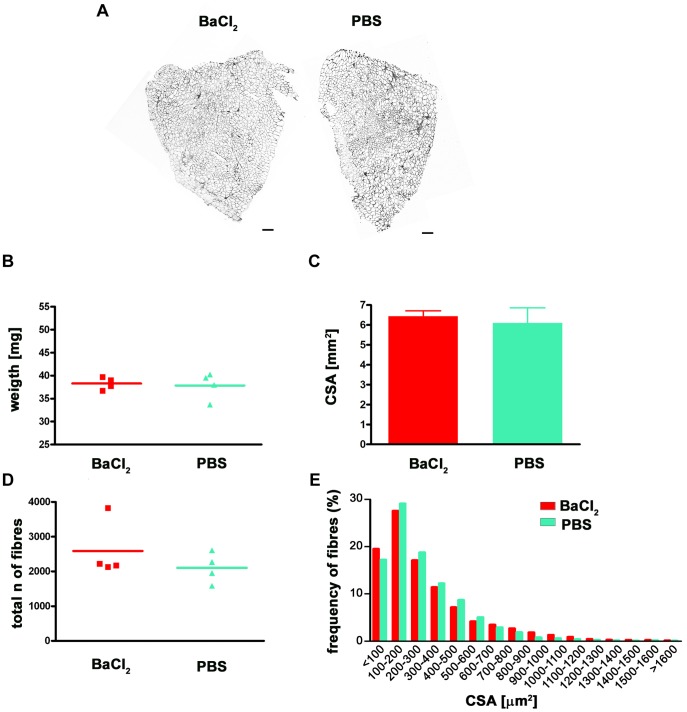
Injection of BaCl2 does not cause muscle hypertrophy. Mdx nude mice (n = 4) had their right TA injected with BaCl_2_ and the left TA with PBS. Laminin-stained transverse sections showed no difference in size between muscles treated in these ways (A). Weights of the muscles were comparable (B) as was the CSA (C). The number of fibres was not significantly different (D) and the distribution of the fibre size was similar (E) (Chi-squared test, p = 0.2261). Size bar = 100 µm.

To ascertain if BaCl_2_ alone caused muscle hypertrophy, the right TA of *mdx* nude mice was injected with BaCl_2_ and the left TA with PBS. Transverse sections of BaCl_2_-injured and PBS-injected TA muscles were similar in size (Figure 2A). This lack of difference was confirmed by a similar weight of muscles treated with either BaCl_2_ or an equal volume of PBS (Figure 2B), a comparable CSA (Figure 2C) and a similar fibre number and distribution of the fibre sizes (Figure 2D and E). From these results, we conclude that BaCl_2_ alone does not promote muscle hypertrophy.

### A Single Donor Myofibre Promotes Muscle Hypertrophy when Injected in Recipient Mouse Muscles

**Figure 3:**
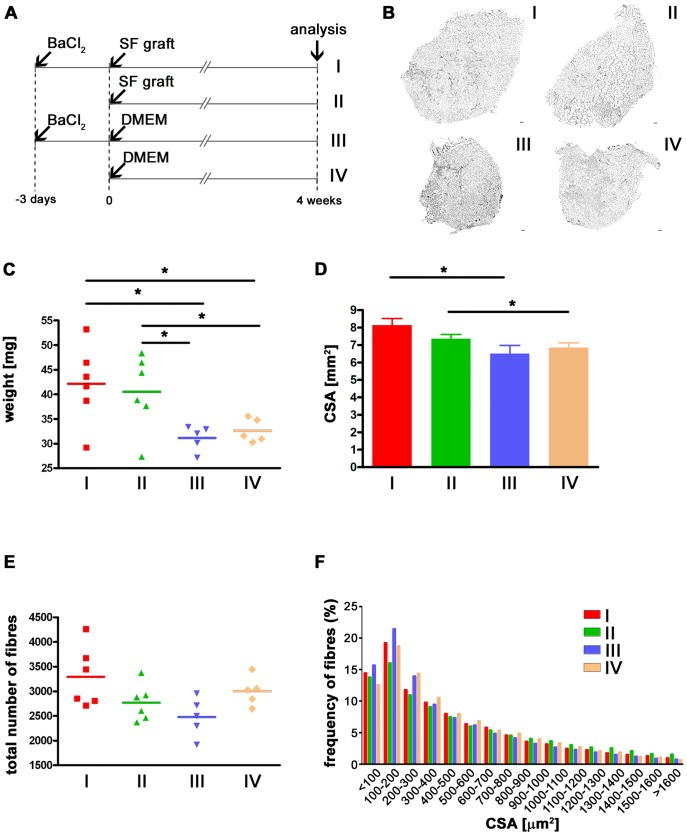
A single donor myofibre injected into recipient mouse muscles promotes muscle hypertrophy. Single fibres were grafted in mdx nude mouse muscles that had either been injured 3 days previously with BaCl_2_ (n = 6) (A-I), or were non-injured (n = 6) (A-II). As a control, DMEM was injected in muscles similarly injured (n = 5) (A-III) or uninjured (n = 5) (A-IV). Representative laminin-stained transverse muscle sections clearly showed that muscles grafted with single fibres (B-I and –II) were macroscopically larger than muscles injected with DMEM (B-III and –IV). This difference was also evident in the weights of the muscles (C). The mean CSA was significantly bigger in muscles in group I compared to III and II compared to IV (D). The number of fibres was not significantly different in any of the cases (E) whilst the distribution of the fibres was changed in muscles injected with single fibres (F) (Chi-squared test: p<0.0001 both when distribution I was compared to III and distribution II was compared to IV). Size bar = 100 µm. *p<0.05.

To identify the cause of the observed muscle hypertrophy, a series of experiments was performed (Figure 3A), in which either a single myofibre isolated from a 3F-*nlacZ*-2E mouse, or DMEM alone was grafted into either BaCl_2_ pre-injured muscles (Figure 3A I, III), or in untreated muscles (Figure 3A II, IV). From a first macroscopic comparison of laminin-stained cryosections, it was evident that muscles grafted with single fibres were bigger than those injected with DMEM (Figure 3B). Moreover, single fibre-grafted muscles were significantly heavier compared to DMEM-injected muscles, despite the absence of donor-derived muscle (Figure 3C). CSAs of pre-injured single fibre-grafted muscles were significantly increased compare to BaCl_2_ pre-injured and DMEM-injected muscles and a similar difference was observed without pre-injuring the muscle (Figure 3D). The number of fibres in the analysed muscles was comparable (Figure 3E) for all the conditions, but the frequency of the fibre size distribution was significantly different, with fewer small fibres and more fibres of larger calibre in muscles injected with a donor fibre (Figure 3F). We therefore conclude that the hypertrophic effect is induced by the injected donor single myofibres, even without pre-injury of the recipient muscles.

### The Hypertrophic Effect is Mediated by the Donor Fibre Rather than Donor Satellite Cells

**Figure 4:**
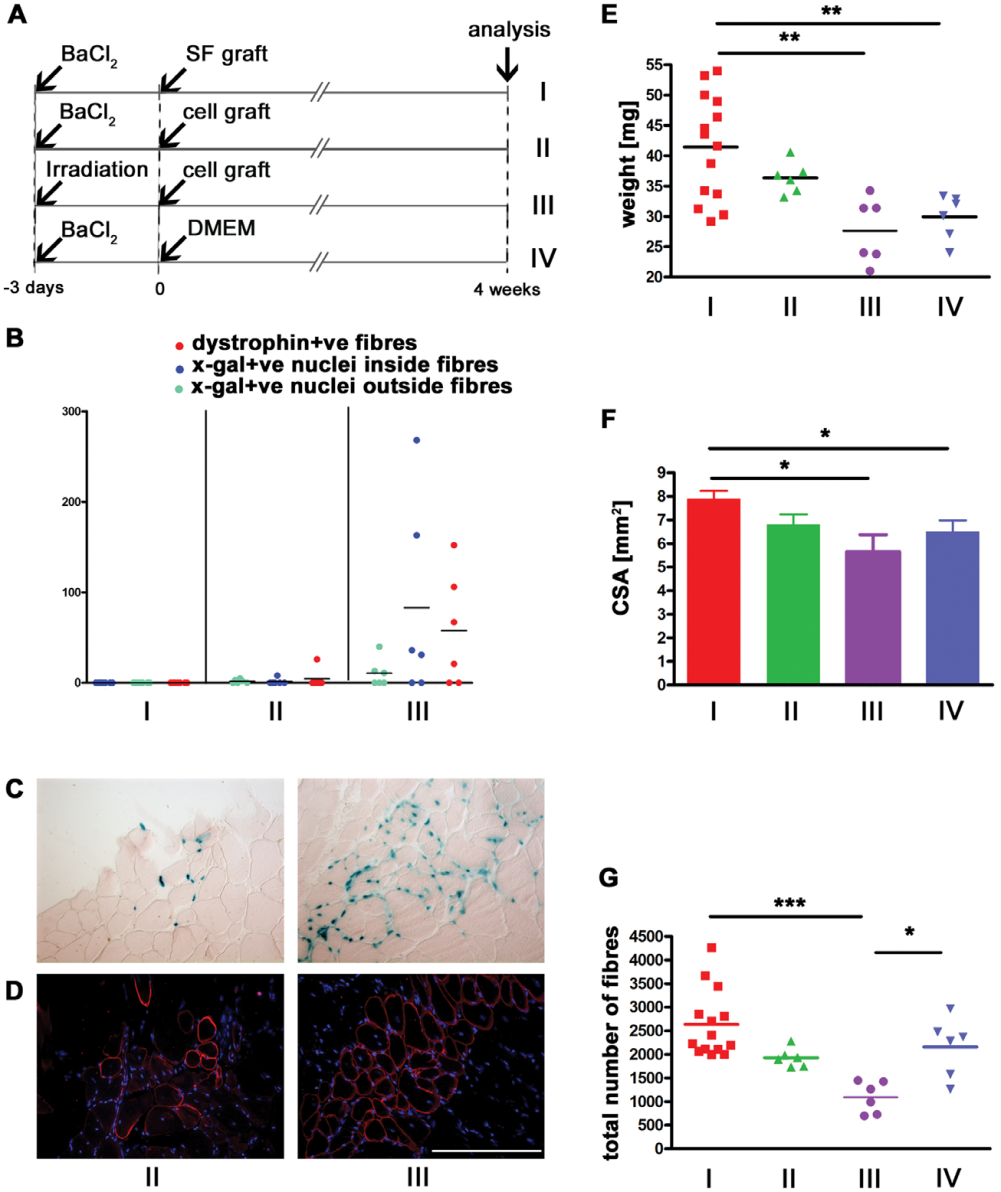
A donor fibre is required for the hypertrophic effect. BaCl_2_-injured muscles were grafted 3 days later with single fibres (n = 8) (A−I), satellite cells (n = 6) (A−II), or DMEM (n = 6) (A−IV); as a control, irradiated muscles were grafted 3 days later with satellite cells (n = 6) (A−III). As fibres and satellite cells were obtained from β-actin-Cre:R26NZG donor mice (n = 2), their in vivo survival and integration in the recipient host muscles outside myofibres could also be determined. This was quantified alongside the presence of donor-derived dystrophin positive fibres (B). As shown by representative pictures, X-gal positive donor-derived nuclei were found in both BaCl_2_-injured (II) and irradiated (III) cell-grafted muscles, inside or nearby the donor-derived dystrophin positive myofibres (C and D respectively). Weights of muscles grafted with fibres (I) were significantly greater than muscles injected with BaCl_2_ and DMEM (IV) or irradiated and cell grafted host muscles (III) (E). This increase in size was mirrored by the increased CSA (F), whilst the total number of fibres was not significantly different from the control (IV) (G). Size bar = 100 µm. *p<0.05; **p<0.01; ***p<0.0001.

As an isolated donor myofibre, bearing its complement of approximately 7 satellite cells [6], grafted into host muscle was able to mediate muscle hypertrophy, we wished to see whether satellite cells removed from their fibre were also capable of causing this effect. We therefore designed a series of experiments where either single fibres, or freshly-stripped satellite cells, were isolated from β-actin-Cre:R26NZG donor mice and grafted into BaCl_2_-treated host mouse muscles. This enabled us to determine whether donor cells had given rise to cells other than skeletal muscle fibres or satellite cells, which might be promoting the host muscle hypertrophy. As a positive control, satellite cells were grafted in pre-irradiated muscles [45] and, as a negative control, BaCl_2_-injured muscles were injected with DMEM (Figure 4A). Quantification of donor-derived muscle and donor-derived nuclei inside and outside myofibres showed that, as expected, fibre formation derived from donor satellite cells was robust in pre-irradiated muscles (58±25 myofibres of donor origin, 83±45 donor-derived myonuclei), with a minority of donor-derived nuclei outside the basal lamina of donor-derived myofibres (11±6) (Figure 4B, C-III, D-III). BaCl_2_-injured and single fibre-grafted muscles not only contained no donor-derived muscle, as previously found (Figure 1B), but also no donor-derived cells outside the basal lamina (Figure 4B). Similarly, satellite cells grafted in BaCl_2_-injured muscles formed few donor-derived fibres (4±4) and the presence of donor-derived nuclei inside and outside the fibres was rare (1±1 and 2±1 respectively) (Figure 4B, C-II, D-II). BaCl_2_–treated muscles injected with single fibres rather than those injected with satellite cells were significantly heavier than either BaCl_2_–treated muscles injected with DMEM, or muscles irradiated and grafted with satellite cells (Figure 4E). The significant increase in CSA in BaCl_2_–treated muscles injected with single fibres mirrored this difference (Figure 4F). Since the total number of fibres in BaCl_2_ pre-injured single fibre-grafted muscles was not significantly increased (Figure 3E and Figure 4G), we conclude that the grafted donor fibre plays a pivotal role in promoting the hypertrophic effect in host muscles.

## Discussion

Evidence that a single grafted donor myofibre can dramatically change host skeletal muscle by contributing robustly to skeletal muscle regeneration came from experiments employing the same *in vivo* system as we used here – fibres from donor genetically-modified wild type mice grafted into pre-irradiated muscles of dystrophin-deficient *mdx* nude mice [6]. Further studies showed that modulation of the host muscle environment is an important requirement for successful donor satellite cell engraftment: not only does the host niche need to be preserved, but also endogenous satellite cells have to be impaired [45]. Such modulation, achieved by irradiating host muscles, permits aged host muscle to be regenerated by donor satellite cells as well as young host muscle [7], [47]. Myotoxins, such as BaCl_2_, notexin and cardiotoxin, have been widely used to cause muscle injury [48], [49]. These destroy myofibres, but myofibre basal lamina, satellite cells, nerves and blood vessels are preserved [48]. In response to the muscle injury, endogenous satellite cells activate, proliferate, migrate and either repair injured fibres, or regenerate new fibres [50], [51]; thus the contribution of transplanted donor cells in competition with efficient host-mediated muscle regeneration is negligible [45]. Among the myotoxins we tested, BaCl_2_ was the only one, when injected 3 days before cell grafting, that promoted significantly more donor-derived muscle formation than in the non-treated host muscles, even though donor muscle formation was 10 times less than in the irradiated grafted muscles [45]. We were therefore interested to see the effect of BaCl_2_ on grafted single fibres, bearing their complement of satellite cells.

We clearly show that, in our model system, donor muscle formation derived from isolated donor myofibres grafted into in BaCl_2_-injured host *mdx* nude muscles is rare and insignificant. However, although they do not give rise to either muscle fibres, or other cell types, within BaCl_2_-treated host muscles, a donor single fibre stimulated host muscle hypertrophy. The number of fibres has not increased, but the diameter of the fibres has, leading to a significant increase in muscle weight. The effect of the grafted isolated fibre on the host muscle is therefore hypertrophy, not hyperplasia, as it is an increase in fibre size rather than number.

Intriguingly, this donor fibre-mediated hypertrophic effect occurred without pre-injury of the host muscle with BaCl_2_, indicating that non-treated *mdx* nude muscles, which would be undergoing some degeneration and regeneration [52]–[54], are also susceptible to this effect. Interestingly, this hypertrophic effect cannot be recapitulated by satellite cells freshly removed from their niche. We speculate that either the donor fibre itself, or components of the satellite cell niche on the donor fibre [45], can signal to the host muscle to evoke its hypertrophy. This is probably a rapid response triggered by the grafting of the fibre, as it occurs even when there is no evidence of survival of either the donor fibre, or the progeny of its satellite cells, 4 weeks after grafting. This could happen in many ways. The crucial pathway that regulates muscle hypertrophy is initiated by binding of IGF1 to the IGF receptor, which then induces activation of Akt/mTOR: this pathway not only leads to inhibition of proteolytic degradation, but also to stimulation of new protein synthesis [55]. However, it has been shown that hypertrophy through Akt/mTOR activation can also be induced independently of activation of IGF receptor: for example, during muscle regeneration, overexpression of Wnt7a, which is a member of the Wnt gene family [56], generates increased number of larger myofibres, inducing expansion of satellite cells, which, when quiescent, express the Wnt7a receptor [57]. This stimulation of hypertrophic myofibre growth is triggered even with minimal induction of regeneration after injection of recombinant Wnt7a factor, through a non-canonical anabolic signalling pathway [58].

Our results show that, even in the presence of a minimal injury created by the needle during single fibre engraftment, the hypertrophic effect is initiated by the donor fibre, but does not occur if medium without a fibre is injected. In addition, the pathway controlling muscle regeneration could be differentially regulated in dystrophic compared to non-dystrophic muscles. We therefore hypothesize that a donor wild type fibre exposes the dystrophic host muscle to growth stimuli that are not normally present within dystrophic muscle: for example, calcineurin signalling, that mediates muscle hypertrophy [59], is aberrant in *mdx* muscles, but, if overexpressed, can ameliorate their regeneration [60].

Our findings have some similarities, but also some differences, to previous work that concluded that isolated fibres grafted into injured mouse muscle have a hypertrophic effect, but that donor satellite cells contributed robustly to muscle fibre regeneration [61]. Similar to our findings, Hall et al. found that neither injury, nor myofiber transplantation alone increases muscle mass. In contrast to our findings, they concluded that the increase in muscle mass was donor satellite cell mediated, as they found, again in stark contrast to our findings, that grafted isolated fibres contributed to robust regeneration within injured host muscles. These discrepancies may be explained by differences in experimental procedures between the two studies. In the experiments that Hall et al. performed, single fibres were grafted after 3–4 hours of incubation in medium containing 15% horse serum at ∼6% O_2_ in the presence of 1.5 nM fibroblast growth factor–2 for 4 to 5 hours. Isolated fibres were then transferred into 40 ml of 1.2% BaCl_2_ and fibres were injected in a volume of 70 µl of 1.2% BaCl_2_ into each host muscle. Hall et al. transplanted 5 donor myofibres per host muscle and used GFP as a marker of muscle and satellite cells of donor origin, whereas we grafted one freshly-isolated fibre per host muscle and used dystrophin and either myosin 3F-*nlacZ*-2E or β-actin-Cre:R26^NZG^ as markers of either muscle fibres or nuclei of donor origin. Hall et al used non-dystrophic, non-immunodeficient host mice (C57Bl/6xDBA2), whereas we used dystrophin deficient, immunodeficient hosts (*mdx* nude) whose muscles had been injured by injection of 25 µl of 1.2% BaCl_2_ 3 days previously.

Our results show that a wild type donor fibre can stimulate the hypertrophic growth of *mdx* muscle without making any direct contribution to the host muscle tissue. How this happens and from which compartment of the fibre the paracrine signalling originates are questions for future investigation. However, that such a simple procedure -merely grafting an isolated muscle fibre- promotes hypertrophy in a dystrophic muscle could have future therapeutic implications.
